# Sterile inflammation in LHON: mitochondrial stress, innate immune signaling, and implications for AAV gene therapy

**DOI:** 10.3389/fimmu.2026.1935135

**Published:** 2026-09-18

**Authors:** Xue Zhang, Aiqin Chang, Yuan Liu, Hong Yu

**Affiliations:** 1 Department of Ophthalmology, The First Affiliated Hospital of Zhengzhou University, Zhengzhou, Henan, China; 2 Bascom Palmer Eye Institute, University of Miami Miller School of Medicine, Miami, FL, United States

**Keywords:** adeno-associated virus, gene therapy, innate immunity, Leber hereditary optic neuropathy, mitochondrial DNA, mitochondrial stress, sterile inflammation

## Abstract

Leber hereditary optic neuropathy (LHON) is a mitochondrial disease caused primarily by pathogenic mitochondrial DNA (mtDNA) variants that impair respiratory chain complex I function. Although bioenergetic failure, oxidative stress, and retinal ganglion cell degeneration are central features of disease pathogenesis, these mechanisms do not fully explain the incomplete penetrance, male predominance, and marked clinical heterogeneity observed in LHON. Emerging evidence suggests that mitochondrial dysfunction can also trigger sterile inflammatory responses through the release of mitochondrial damage-associated molecular patterns (DAMPs), including mtDNA and reactive oxygen species, and through impaired mitophagic clearance. These signals activate innate immune pathways, including cGAS-STING, TLR9, and the NLRP3 inflammasome, potentially contributing to neuroinflammation, glial activation, and secondary neuronal injury. The relevance of immune signaling has gained further attention with the development of AAV-based gene therapies for LHON, where treatment-associated ocular inflammation has emerged as a clinically important challenge. In this review, we examine the evidence linking mitochondrial dysfunction to innate immune activation in LHON, discuss the immunological mechanisms underlying disease progression and gene therapy-associated inflammation, and highlight emerging opportunities for biomarker development, immunomodulatory intervention, and improved therapeutic design. We propose that LHON should be viewed not only as a disorder of mitochondrial bioenergetics but also as a neuroinflammatory disease shaped by the interplay between mitochondrial stress and immune signaling.

## 1 Introduction

Leber hereditary optic neuropathy (LHON) is a maternally inherited mitochondrial optic neuropathy characterized by selective degeneration of retinal ganglion cells (RGCs) and their axons, causing subacute, painless central vision loss that is often sequential but typically becomes bilateral within weeks to months (1). Most genetically confirmed cases are attributable to primary mitochondrial DNA (mtDNA) missense variants affecting complex I (NADH:ubiquinone oxidoreductase), including m.11778G>A (*MT-ND4*), m.3460G>A (*MT-ND1*), and m.14484T>C (*MT-ND6*) (1, 2). These variants disrupt electron transport and support the classification of LHON as a complex I–linked mitochondrial optic neuropathy (1, 2).

The traditional pathogenic model of LHON centers on complex I dysfunction. This defect impairs oxidative phosphorylation, reduces ATP production, and increases reactive oxygen species (ROS), leading to energetic stress and oxidative injury in RGCs (1). However, this mechanism alone does not fully explain why the disease develops in some mutation carriers but not in others, or why clinical expression varies so widely. LHON is marked by incomplete penetrance and a clear male predominance, indicating that many mutation carriers remain unaffected while others develop visual loss (1). Earlier studies estimated a higher lifetime risk of visual loss in male carriers than in female carriers, although more recent population-based studies suggest that penetrance may be lower and varies across cohorts (3). Environmental exposures influence disease conversion. Smoking has a strong and consistent association with visual loss in LHON mutation carriers (4). These findings indicate that the primary bioenergetic defect is necessary but not sufficient for disease expression. Clinical conversion is likely shaped further by genetic background, sex-related biological factors, and environmental modifiers.

Bibliometric mapping shows that LHON research has evolved from early studies of mitochondrial genetics, pedigrees, and clinical manifestations toward retinal imaging, disease mechanisms, clinical trials, and gene therapy (**Figure 1**). Citation-burst analysis further identifies gene therapy, retinal ganglion cells, prevalence, clinical trials, epidemiology, LHON, and idebenone as active or recently emerging research topics through 2025 (**Figure 2**). These trends place mitochondrial mechanisms and therapeutic translation at the center of current LHON research.

**Figure 1:**
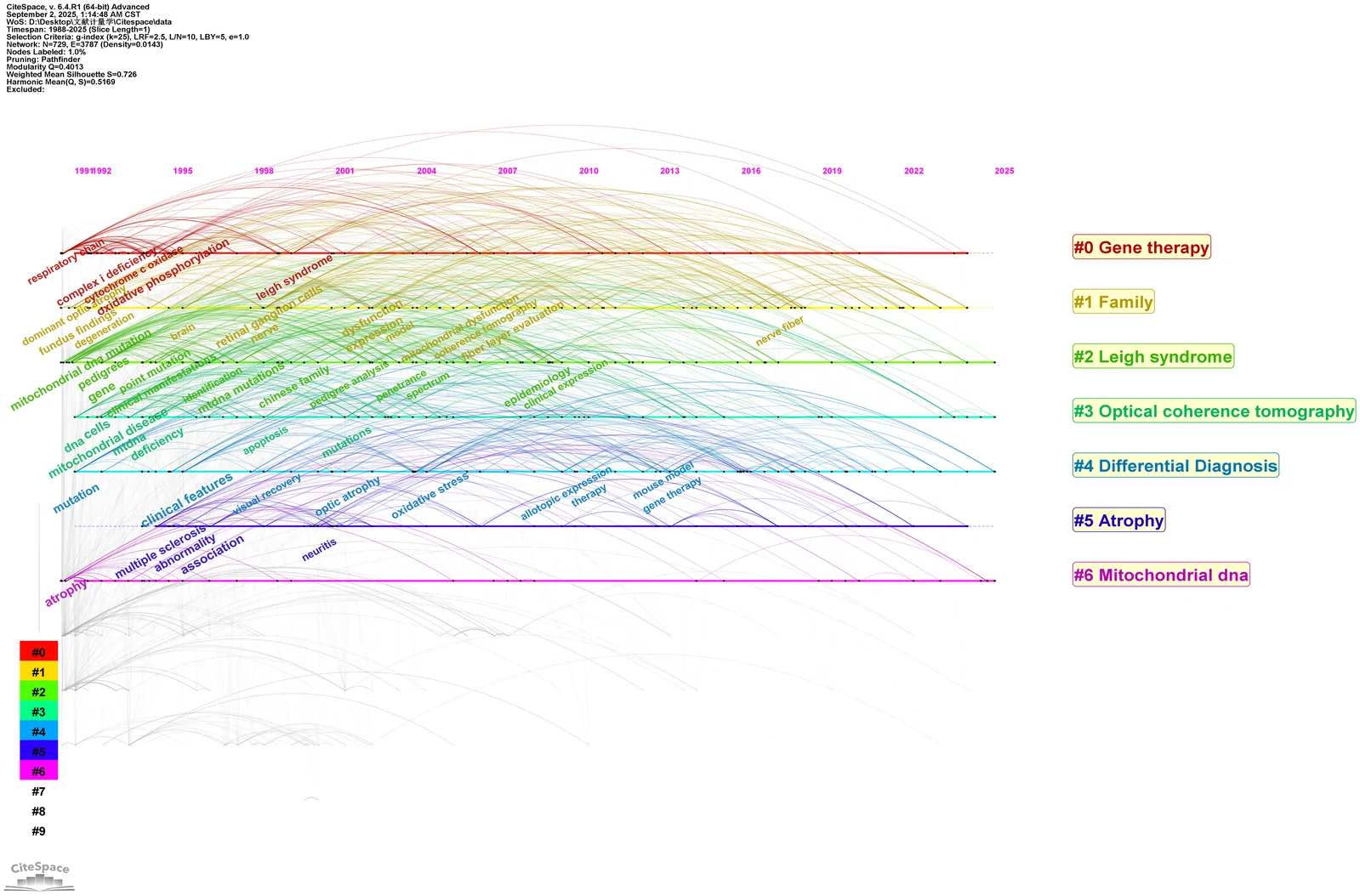
Timeline view of keyword clusters in Leber hereditary optic neuropathy (LHON) research from 1988 to 2025. The timeline shows the evolution of major keyword clusters over time. Colored lines represent distinct clusters, and nodes indicate representative keywords in specific periods. Major clusters include gene therapy, family, Leigh syndrome, optical coherence tomography, differential diagnosis, atrophy, and mitochondrial DNA, reflecting the thematic development of the field.

**Figure 2:**
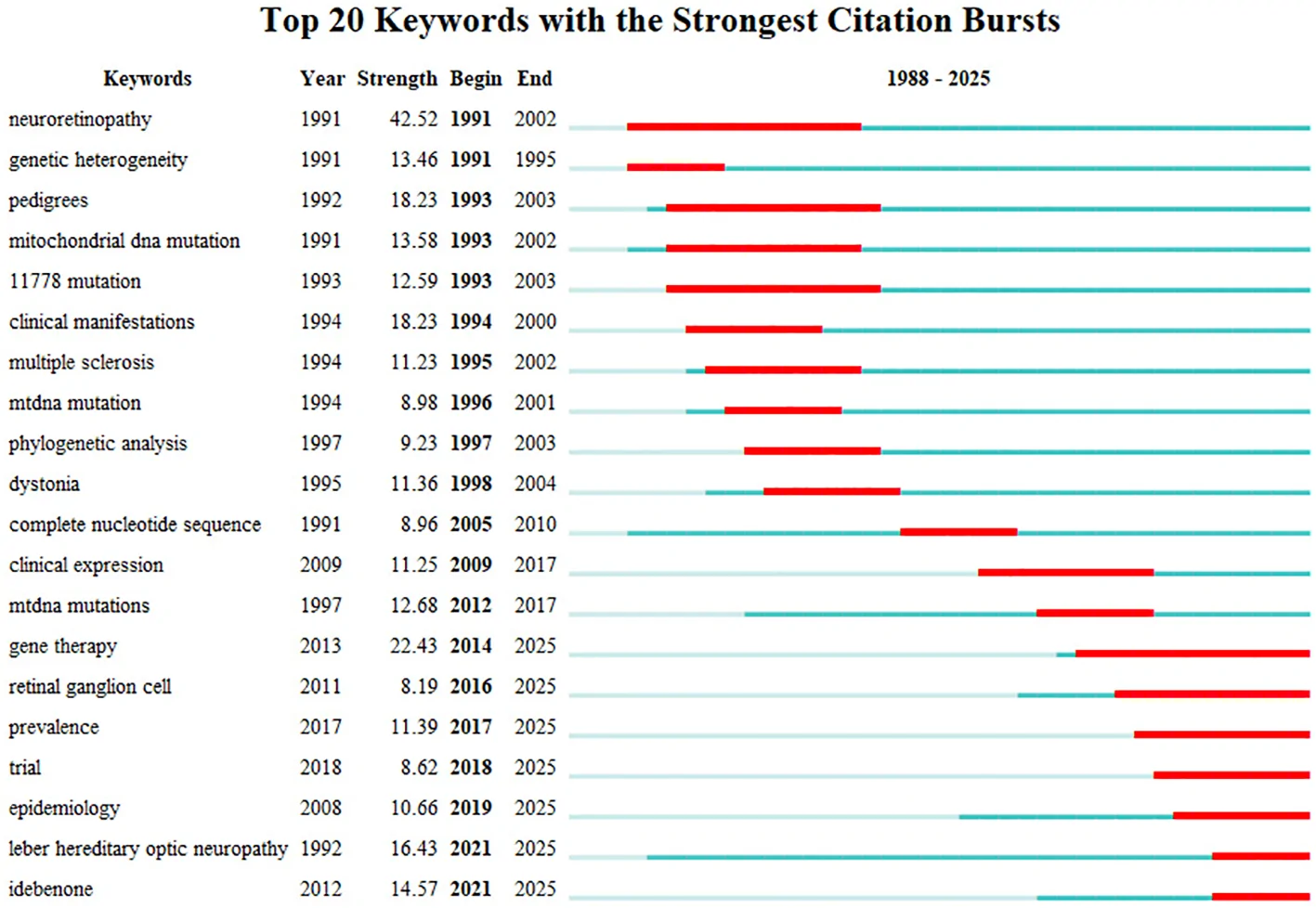
Top 20 keywords with the strongest citation bursts in LHON research from 1988 to 2025. The figure shows the top 20 keywords with the strongest citation bursts in LHON research. Red bars indicate periods of increased research attention, whereas blue lines represent the full observation period from 1988 to 2025. Early burst keywords included neuroretinopathy, genetic heterogeneity, mitochondrial DNA mutation, and 11778 mutation. Recent burst keywords included gene therapy, retinal ganglion cell, prevalence, trial, epidemiology, Leber hereditary optic neuropathy, and idebenone. These keywords reflect current research frontiers and emerging trends in the field.

In parallel, immune and inflammatory pathways have become increasingly relevant to interpretations of disease progression and therapeutic response. In experimental systems, mitochondrial dysfunction promotes the generation and abnormal redistribution of mitochondrial damage-associated molecular patterns (mtDAMPs), linking organelle stress to innate immune signaling (5). This perspective has gained further clinical relevance with the development of intravitreal AAV-based gene therapy, in which ocular inflammation has emerged as an important consideration alongside therapeutic efficacy (6).

This focused narrative review examines how mitochondrial stress, mtDNA-related innate immune signaling, and treatment-associated immune responses may shape disease expression and therapeutic outcomes in LHON. Where relevant, we also consider LHON–multiple sclerosis overlap as a rare clinical phenotype illustrating the possible interaction between mitochondrial vulnerability and immune susceptibility. The bibliometric findings provide the broader research context for the mechanistic and translational synthesis developed below.

To identify relevant publications and generate the bibliometric analyses presented in **Figures 1**, **2**, a systematic search of the Web of Science Core Collection (WoSCC) was conducted for publications from 1988 to 2025. The search terms included “Leber hereditary optic neuropathy,” “Leber’s hereditary optic neuropathy,” and “LHON.” Publications were eligible if they were indexed in WoSCC during the specified period and directly addressed the genetics, pathogenesis, clinical characteristics, diagnosis, imaging, or treatment of LHON. Duplicate records and publications unrelated to LHON were excluded. A total of 1,609 publications were included in the final bibliometric dataset. CiteSpace, VOSviewer, and R were used to analyze publication trends, citation networks, international collaboration, keyword co-occurrence, temporal keyword clusters, and citation bursts.

## 2 The trigger: mitochondria as sources of DAMPs

Primary mtDNA mutations in LHON, most of which affect genes encoding respiratory chain complex I subunits, have consequences beyond impaired ATP production. They also drive persistent mitochondrial stress, disrupt organelle homeostasis, and increase membrane fragility, thereby facilitating abnormal exposure or release of mitochondrial constituents (7). Consistent with their endosymbiotic origin, mitochondria retain several bacterial-like molecular features (8), including hypomethylated, CpG-containing mtDNA and N-formyl peptides initiated by N-formylmethionine (9). When released under conditions of mitochondrial disequilibrium, these molecules can be recognized by the innate immune system as signals of microbial invasion. They then act as mtDAMPs, engage pattern-recognition receptors, and initiate a robust sterile inflammatory response even in the absence of an exogenous pathogen (9).

### 2.1 Sources and routes of mtDNA release

The cytosolic release of oxidized mtDNA represents one of the clearest mechanisms linking mitochondrial injury to inflammatory signaling (10–12). mtDNA fragmentation further facilitates its escape and enhances its immunogenicity. Notably, this process is not confined to terminal cell breakdown. mtDNA is not a static molecule but undergoes a regulated life cycle, with degradation occurring throughout the cellular life cycle via multiple pathways (13–15). Cells coordinate mtDNA trafficking and compartmentalization to balance degradation with immune tolerance. When this balance is disrupted under stress, inflammatory responses can be triggered, suggesting that mitochondrial genome leakage may function as an active stress response rather than a mere passive consequence of cell death (16, 17).

Under sublethal oxidative stress, voltage-dependent anion channel 1 (VDAC1) in the outer mitochondrial membrane can oligomerize and form large pores, allowing the escape of short mtDNA fragments into the cytosol. This process can activate type I interferon (IFN-I) signaling and promote innate immune priming (16). Oxidized mtDNA within mitochondria may undergo nuclease-mediated fragmentation, generating fragments of approximately 500–650 base pairs. These fragments can then exit through channels involving both the mitochondrial permeability transition pore (mPTP) and VDAC, thereby triggering downstream inflammasome activation and interferon responses (18).

During apoptosis, mtDNA also reaches the cytosol through a distinct but related route. BAX- and BAK-mediated MOMP forms large outer membrane pores and can be accompanied by inner membrane herniation, thereby enabling the release of mitochondrial matrix components, including mtDNA, into the cytosol (17). However, mtDNA release alone does not necessarily dictate the inflammatory outcome. When caspase activity is preserved, apoptosis remains relatively immunologically silent; when caspase activation is blocked, however, cytosolic mtDNA can activate the cGAS–STING axis and drive a stronger type I interferon response (19). These observations highlight that both the route of mtDNA escape and the apoptotic context in which it occurs are critical determinants of innate immune activation.

### 2.2 Mitophagy impairment

The generation of mtDAMPs alone is unlikely to fully explain the persistent low-grade inflammatory state hypothesized in LHON. Under physiological conditions, mitochondrial quality-control systems, especially mitophagy, continuously eliminate dysfunctional mitochondria and their associated nucleic acids, thereby limiting endogenous danger signals at their source (20). When this barrier is compromised, transient organelle injury may give rise to sustained innate immune activation. Experimental studies show that defective autophagic clearance permits intracellular accumulation of escaped mtDNA, thereby engaging DNA-sensing pathways, including Toll-like receptor 9 (TLR9)-dependent inflammatory signaling (21). Loss of key autophagy regulators, including LC3B and Beclin 1, similarly promotes the persistence of damaged mitochondria, facilitates mtDNA translocation into the cytosol, and amplifies NLR family pyrin domain-containing 3 (NLRP3) inflammasome-mediated release of interleukin-1 beta (IL-1β) and interleukin-18 (IL-18) (20). In addition, declining mitophagic flux has been linked to increased cytosolic mtDNA leakage and stronger STING activation, while interferon-driven mitochondrial stress may further enhance mtDNA release, creating a self-reinforcing mitophagy-mtDNA-STING loop that sustains chronic inflammation (22).

Evidence from LHON models broadly supports this framework. In cybrid and patient-derived cellular systems harboring pathogenic LHON-associated mtDNA variants, abnormal autophagic or mitophagic responses have been associated with reduced PTEN-induced kinase 1 (PINK1)/Parkin signaling, altered LC3 and p62 profiles, accumulation of dysfunctional mitochondria, increased ROS levels, and greater apoptotic susceptibility (23, 24). These observations point to a disturbance in mitochondrial quality control in LHON, although its exact nature may vary across models and may reflect insufficient, dysregulated, or maladaptive mitophagy rather than a single, uniform defect. Human induced pluripotent stem cell (iPSC)-derived LHON retinal organoids further extend this concept to a retinal setting, as they reproduce RGC loss, reduced ATP production, mitochondrial dysfunction, and decreased mitophagy (25). These findings identify impaired mitochondrial quality control as a mechanistic link between mitochondrial injury and persistent mtDNA-related innate immune signaling in LHON models.

### 2.3 Receptor sensing and signal amplification

Once released into the cytosol or extracellular space, mitochondrial components are recognized by pattern-recognition receptors. This recognition connects mitochondrial damage with innate immune activation and inflammatory injury.

The cyclic GMP–AMP synthase–stimulator of interferon genes (cGAS–STING) pathway is an established cytosolic DNA-sensing pathway. cGAS recognizes cytosolic double-stranded DNA, including released mtDNA, and produces cyclic GMP–AMP (cGAMP). cGAMP activates STING, which then signals through TANK-binding kinase 1 (TBK1), interferon regulatory factor 3 (IRF3), and nuclear factor κB (NF-κB). This process induces type I interferon production and other inflammatory genes (26–28). In mouse models of glaucoma and retinal ischemia/reperfusion injury, microglial cGAS–STING activation promotes neuroinflammation and retinal ganglion cell loss. Genetic or pharmacologic inhibition of this pathway reduces inflammation and preserves visual function (29, 30). These findings establish the role of cGAS–STING signaling in experimental retinal injury. However, direct evidence of cGAS–STING activation in LHON remains limited.

The NLRP3 inflammasome is a well-established mediator of inflammatory responses to mitochondrial stress. Excessive reactive oxygen species production, mitochondrial damage, and cytosolic oxidized mtDNA promote NLRP3 inflammasome activation. This activation leads to caspase-1 engagement and the maturation of interleukin-1β and interleukin-18 (10, 18, 31). NLRP3 signaling can also aggravate mitochondrial injury, creating a cycle of mitochondrial damage and inflammatory amplification. These mechanisms are well established in experimental systems, but their direct contribution to LHON has not yet been demonstrated.

Toll-like receptor 9 (TLR9) provides another route for mtDNA recognition. TLR9 is an endosomal DNA receptor that recognizes unmethylated CpG-rich DNA and activates myeloid differentiation primary response 88 (MyD88)–dependent NF-κB signaling (21, 32). This pathway increases the expression of inflammatory cytokines and connects extracellular or endosomal mtDNA with innate immune activation. Evidence from non-LHON experimental systems supports this mechanism (33), but direct evidence of TLR9 activation in patients with LHON is still lacking.

### 2.4 Stress response modulation of mitochondrial innate immunity

Beyond releasing mtDAMPs, mitochondrial dysfunction can alter innate immune thresholds through retrograde stress programs. Genome-wide CRISPR screening and genetic perturbation studies have shown that mitochondrial stress activates OMA1–dependent DELE1 cleavage, enabling cytosolic DELE1 to engage HRI and induce eIF2α phosphorylation and ATF4 translation (34, 35). Separately, loss of CLPP causes mtDNA instability and cytosolic leakage, leading to cGAS–STING-dependent type I interferon signaling in cultured cells and mouse tissues (36). Pyrimidine imbalance induced by YME1L deficiency similarly promotes mtDNA release and cGAS–STING-TBK1 activation, including an inflammatory response in the mouse retina (37). These findings establish experimental cross-talk between mitochondrial stress adaptation, metabolic homeostasis, and mtDNA sensing, although whether these pathways modify inflammation or RGC loss in LHON remains unknown.

### 2.5 Glial crosstalk in LHON

The proinflammatory cytokines and chemotactic signals generated by these receptor pathways suggest that RGC degeneration in LHON is not solely a cell-autonomous process. Instead, complex I dysfunction imposes sustained bioenergetic and metabolic stress on vulnerable RGCs, promoting the release of danger signals that reshape the local retinal immune microenvironment. In this setting, a primary mitochondrial defect may trigger a secondary inflammatory response, potentially establishing a feed-forward cycle of neuroinflammatory damage.

As the resident immune cells of the retina, microglia may become activated before overt neuronal loss becomes morphologically apparent. Under stress conditions, they can shift from a surveillant state toward inflammatory and phagocytic phenotypes. A key unresolved question in early LHON is whether microglial activation is initially protective by clearing debris and maintaining tissue homeostasis, or becomes harmful by eliminating stressed but viable neurons, and thereby accelerating neurodegeneration. This possibility is biologically plausible because microglia can mediate phagocytosis of live neurons, and complement-dependent microglial elimination of RGCs has been demonstrated in the developing retina (38, 39).

Reactive Müller glia may also exert a dual effect. During acute stress, Müller cell gliosis can preserve glutamate homeostasis and provide antioxidant and trophic support. With persistent stress, however, this response may adopt a proinflammatory and neurotoxic phenotype, increasing the mitochondrial burden on injured RGCs and reinforcing a hostile retinal microenvironment (40).

Human LHON optic nerve pathology reveals marked axonal loss with fibrotic and fibrillary glial remodeling, consistent with structural reorganization during neurodegeneration (41). Experimental models of optic nerve injury further indicate that astrocyte reactivity is not merely an epiphenomenon but actively influences neuronal survival and functional outcomes (42). Collectively, reciprocal RGC-glial signaling may constitute a pathological feed-forward loop that determines whether mitochondrial injury remains cell-autonomous or propagates into irreversible tissue-level neurodegeneration. **Figure 3** summarizes the proposed sequence linking primary mtDNA defects, mtDAMP accumulation, innate immune activation, glial remodeling, and retinal ganglion cell degeneration in LHON.

**Figure 3:**
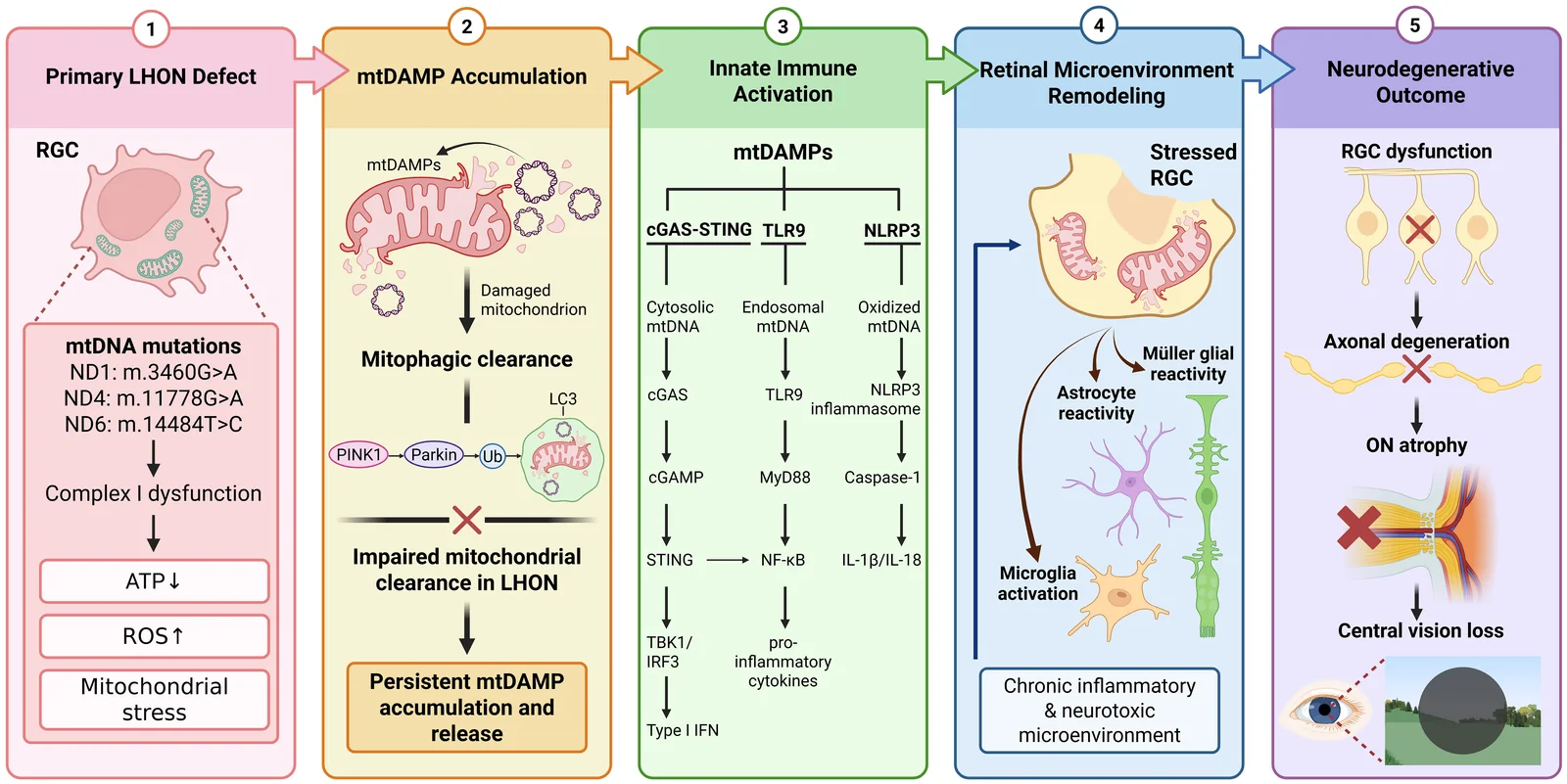
Proposed mechanism linking mitochondrial damage-associated molecular patterns (mtDAMPs) to innate immune activation and neurodegeneration in LHON. Primary LHON-associated mtDNA mutations, most often affecting complex I subunits, reduce ATP production, increase reactive oxygen species, and induce persistent mitochondrial stress in retinal ganglion cells (RGCs). Damaged mitochondria may release mtDAMPs, particularly mtDNA, while impaired mitophagy may promote their accumulation. These signals can activate cGAS–STING, TLR9, and the NLRP3 inflammasome, leading to NF-κB- and interferon-related signaling, sterile inflammation, glial activation, RGC injury, axonal degeneration, optic nerve atrophy, and progressive central vision loss. Source: Biorender.com.

## 3 LHON–MS overlap: a rare clinical boundary phenotype

A small subset of LHON mutation carriers develops an inflammatory demyelinating central nervous system (CNS) syndrome that meets diagnostic criteria for multiple sclerosis (MS) or a closely related MS-like disorder, commonly referred to as LHON–MS overlap or Harding disease (43–46). This rare phenotype should not be interpreted as evidence that autoimmune inflammation is a general feature of LHON. Rather, it provides a clinical example in which mitochondrial vulnerability may intersect with additional host immune susceptibility to produce a broader neuroinflammatory and demyelinating phenotype in selected individuals (47, 48).

A recent review identified 88 reported cases of LHON–MS overlap through July 2024, with a marked female predominance and m.11778G>A remaining the most common mtDNA variant. This pattern is notable because classical LHON is typically male predominant, whereas the overlap phenotype shows a different sex distribution. However, because the available evidence is largely case-based, it does not establish population prevalence or a causal relationship between LHON mutations and MS. These observations support the possibility that immunogenetic background contributes to phenotypic divergence in a minority of carriers, but they should not be generalized to classical LHON (49).

### 3.1 HLA-related immune susceptibility in the LHON–MS overlap phenotype

Available evidence does not support HLA-DRB1*15:01 or other conventional HLA class I or class II alleles as general risk factors for classical LHON or LHON-related visual failure (1, 50). In population-based genetic studies, Govan and colleagues examined 79 LHON families carrying pathogenic mtDNA mutations and found no significant association between HLA-DR genotype and disease expression in affected versus unaffected carriers (51). Studies addressing classical HLA class I loci, including HLA-A and HLA-B, were likewise largely negative, arguing against the hypothesis that conventional HLA alleles directly determine the development of LHON optic neuropathy (1, 51).

In Harding disease, however, the contribution of HLA may be more complex. Earlier case-based observations suggested a tendency toward enrichment of the DR2/DR15-DQ6 haplotype in patients with LHON–MS overlap. Consistent with this pattern, Govan and colleagues reported that DR15 was present in 31% of women with LHON–MS, compared with 11% of female carriers without MS manifestations. Although these findings are not sufficient on their own to establish causality, they are biologically notable because DR15 represents the strongest and most consistently replicated HLA risk background in MS. Together, these observations support the possibility that, in a subset of LHON carriers, mitochondrial vulnerability may converge with an MS-associated immunogenetic background, contributing to an overlap phenotype with neuroinflammatory and demyelinating features (51–53). Imaging observations of MS-like white matter abnormalities in some female LHON carriers are consistent with this possibility, although these findings do not establish a generalized autoimmune predisposition in women with classical LHON (51, 52).

Mechanistically, HLA-DRB1*15:01 could influence adaptive immune responses through antigen presentation and T-cell repertoire selection, as established in MS rather than directly demonstrated in LHON–MS (53–55). Mitochondrially encoded peptides, stress-modified mitochondrial proteins, or other altered self-antigens may provide candidate antigens under permissive conditions, while impaired clearance of apoptotic or myelin debris may prolong antigen availability (53, 56, 57). However, these HLA-restricted adaptive mechanisms should be distinguished from mtDNA-driven sterile inflammation, which involves innate nucleic-acid sensing rather than peptide-specific autoimmunity. Similarly, the interaction between HLA-DRB1*15:01 and MERTK reported in MS may offer a mechanistic analogy, but its relevance to LHON–MS remains to be directly tested (55).

HLA-related immune background does not appear to cause LHON or determine visual conversion. At most, it may modify susceptibility to an MS-like autoimmune phenotype in a genetically predisposed minority. The overlap phenotype therefore illustrates how mitochondrial vulnerability may be modified by additional immune susceptibility in selected carriers, but it should not be used as evidence that adaptive autoimmunity is a general mechanism of classical LHON.

### 3.2 Mitochondrial vulnerability as a possible amplifier of inflammatory injury

One possible explanation for the overlap phenotype is that pre-existing mitochondrial dysfunction lowers cellular tolerance to inflammatory stress in individuals with additional immune susceptibility. Oligodendrocytes have high bioenergetic demands because myelin formation and maintenance depend heavily on mitochondrial oxidative metabolism (58, 59). Complex I dysfunction could therefore reduce their metabolic reserve and increase their vulnerability to nitric oxide, oxidative stress, and other inflammatory mediators, potentially facilitating demyelinating injury (60–62). However, direct evidence that LHON-associated complex I dysfunction lowers the inflammatory threshold of oligodendrocytes in patients with LHON–MS is currently lacking; this remains a mechanistic hypothesis extrapolated primarily from MS and experimental models.

Separately, mitochondrial injury can activate sterile innate immune pathways through the release of mtDNA and other mtDAMPs, as discussed in Section 2. In the overlap phenotype, such innate signaling could provide a permissive inflammatory background, but it should not be equated with HLA-restricted autoimmunity. Direct evidence for a self-sustaining mtDNA–immune amplification loop in patients with LHON–MS remains limited (62).

Barrier dysfunction represents another plausible, but indirect, amplification mechanism. Evidence from MS and experimental neuroinflammation indicates that mitochondrial oxidative stress can weaken blood–brain barrier or blood–retinal barrier integrity and facilitate immune-cell entry (63–67). Whether LHON-associated mitochondrial stress independently produces comparable barrier dysfunction in patients with LHON–MS has not been established. This pathway should therefore be regarded as a testable extrapolation rather than a confirmed feature of the overlap syndrome.

LHON–MS overlap is best viewed as a rare boundary phenotype rather than as a model applicable to the broader LHON population. It suggests that mitochondrial vulnerability may interact with HLA-associated adaptive immune susceptibility and CNS inflammatory mechanisms in selected carriers, but it does not establish inflammation as a necessary downstream consequence of LHON mutations. Clinically, careful distinction among classical LHON, MS-associated optic neuritis, and true overlap phenotypes remains essential, with MRI and cerebrospinal fluid studies supporting diagnosis and management (47, 48, 50).

This distinction also clarifies the transition to gene therapy. The autoimmune and demyelinating mechanisms considered in LHON–MS involve a rare host phenotype, whereas the inflammation discussed in the following section is an iatrogenic response to exogenous AAV delivery that may occur across a broader treated LHON population. Although both settings involve immune activation, their initiating triggers, immune components, and clinical implications should not be conflated.

## 4 Inflammation in AAV gene therapy

### 4.1 The clinical reality

Lenadogene nolparvovec is an investigational recombinant AAV2/2 vector that delivers a nuclear-encoded wild-type ND4 sequence to retinal ganglion cells by intravitreal injection. Across its clinical development program, intraocular inflammation has been common but has usually been mild, confined to the anterior chamber or vitreous, and responsive to corticosteroid treatment. In the 5-year follow-up of RESCUE and REVERSE, inflammation during the first 2 years occurred in 49 of 62 treated eyes (79.0%) and 5 of 62 sham-treated eyes (8.1%). Few new events were recorded between years 2 and 5 (6). In REFLECT, inflammation occurred in 70.7% of treated eyes and 10.2% of placebo-treated eyes despite a 28-day course of oral corticosteroids started 2 days before injection. Most events were mild or moderate. Posterior inflammation was uncommon and consisted of three cases of retinal vasculitis that resolved after treatment without reported sequelae (68). A pooled analysis of five studies found inflammation in 75.6% of treated eyes. Most events were anterior chamber inflammation or vitritis and resolved with topical corticosteroids (69). These findings support the view that ocular inflammation is a recurrent, treatment-associated feature of this platform, even when overall tolerability remains favorable.

Evidence for a dose effect is suggestive but not uniform. REVEAL included four dose levels ranging from 9 × 10^9^ to 1.8 × 10^11^ vector genomes (vg) per eye. A secondary analysis of the first 96 weeks did not find a correlation between the composite ocular inflammation score and vector dose (70). However, the longer follow-up showed more marked inflammation in the 1.8 × 10^11^ vg/eye cohort. The data safety monitoring board considered inflammation dose-limiting and selected 9 × 10^10^ vg/eye for the phase 3 studies (71). These observations support a dose-related safety signal, but they do not establish a consistent dose-response curve because each REVEAL cohort included only three participants. The larger pooled analysis also cannot resolve this question because 95.2% of patients received 9 × 10^10^ vg/eye (69). Studies of other intravitreal AAV products have shown clearer dose-related inflammation, but direct comparisons with LHON trials are limited by differences in capsid, vector genome, injection volume, corticosteroid regimen, and inflammation grading.

### 4.2 Mechanistic dissection

The vector genome is an established source of innate immune activation. Unmethylated CpG motifs in AAV DNA can be detected by Toll-like receptor 9 in endosomes. TLR9 then signals through MyD88 and activates interferon- and NF-κB-associated pathways. This response can increase local cytokine production and antigen presentation (72–74). Experimental studies show that CpG depletion or the addition of TLR9-inhibitory sequences can reduce inflammation and T-cell infiltration and can preserve transgene expression (72, 75, 76). AAV capsid dose also matters. In nonhuman primates, removal of empty capsids reduced inflammation after intravitreal injection while improving transduction efficiency. These findings show that total capsid exposure contributes to the ocular inflammatory response (77).

In experimental AAV models, adaptive immune activation follows the early innate response. Pre-existing anti-AAV antibodies can reduce intravitreal transduction, and ocular administration can increase systemic antibody titers (78–83). Capsid-derived peptides can also be presented to CD8-positive T cells (74, 84). However, findings from systemic or nonocular AAV models should not be applied directly to the eye. In REVEAL, systemic anti-AAV2 humoral and cellular responses were limited and were not associated with the ocular inflammation score. Baseline neutralizing antibody titers also did not predict inflammation (70). These findings indicate that the common ocular events after lenadogene nolparvovec are mainly local and are not explained by the systemic immune measures used in that study.

The immunogenicity of the ND4 transgene is less clear. Lenadogene nolparvovec encodes a recoded human wild-type ND4 protein rather than a foreign reporter protein. The protein is translated in the cytoplasm and then targeted to mitochondria. In theory, allotopic expression, altered protein processing, or presentation of ND4-derived peptides could produce an immune response in some patients. However, no published human study has shown ND4-specific antibodies or ND4-reactive T cells after treatment. The immune assays used in REVEAL focused mainly on the AAV2 capsid rather than the ND4 protein (70, 71). ND4 immunogenicity should therefore be presented as a testable possibility, not as an established cause of inflammation. Future trials should include ND4-specific T-cell assays and, when feasible, analysis of intraocular cytokines and immune cells.

### 4.3 Relationship between inflammation and visual outcomes

Whether inflammation alters the visual response to AAV-ND4 therapy remains uncertain. None of the published lenadogene nolparvovec trials was designed or powered to determine whether the occurrence, duration, or severity of inflammation predicts best-corrected visual acuity (BCVA). Most inflammatory events were mild, responded to treatment, and did not leave recognized structural or visual sequelae (6, 68, 69). Visual improvement was maintained for up to 5 years despite the high frequency of early inflammation (6). In REFLECT, bilaterally treated patients had inflammation of similar frequency and severity to unilaterally treated patients, while the average visual outcomes favored bilateral treatment (68). These observations do not suggest a large loss of efficacy from the mild events reported in these trials. However, they do not exclude smaller effects on transduction or retinal ganglion cell survival.

The proposed interaction between mitochondrial stress and AAV-induced inflammation also remains hypothetical. Mitochondrial dysfunction may lower the tolerance of retinal ganglion cells to inflammatory stress, while the AAV genome and capsid provide an additional immune stimulus. This two-hit model is biologically plausible, but there is no direct evidence that untreated LHON eyes have a persistent inflammatory state that increases the response to a given vector dose. It should be tested by relating baseline mitochondrial or inflammatory biomarkers to post-injection inflammation, transgene expression, retinal structure, and visual outcomes. **Figure 4** summarizes the established immune responses to intraocular AAV exposure and the proposed contribution of LHON-related mitochondrial stress.

**Figure 4:**
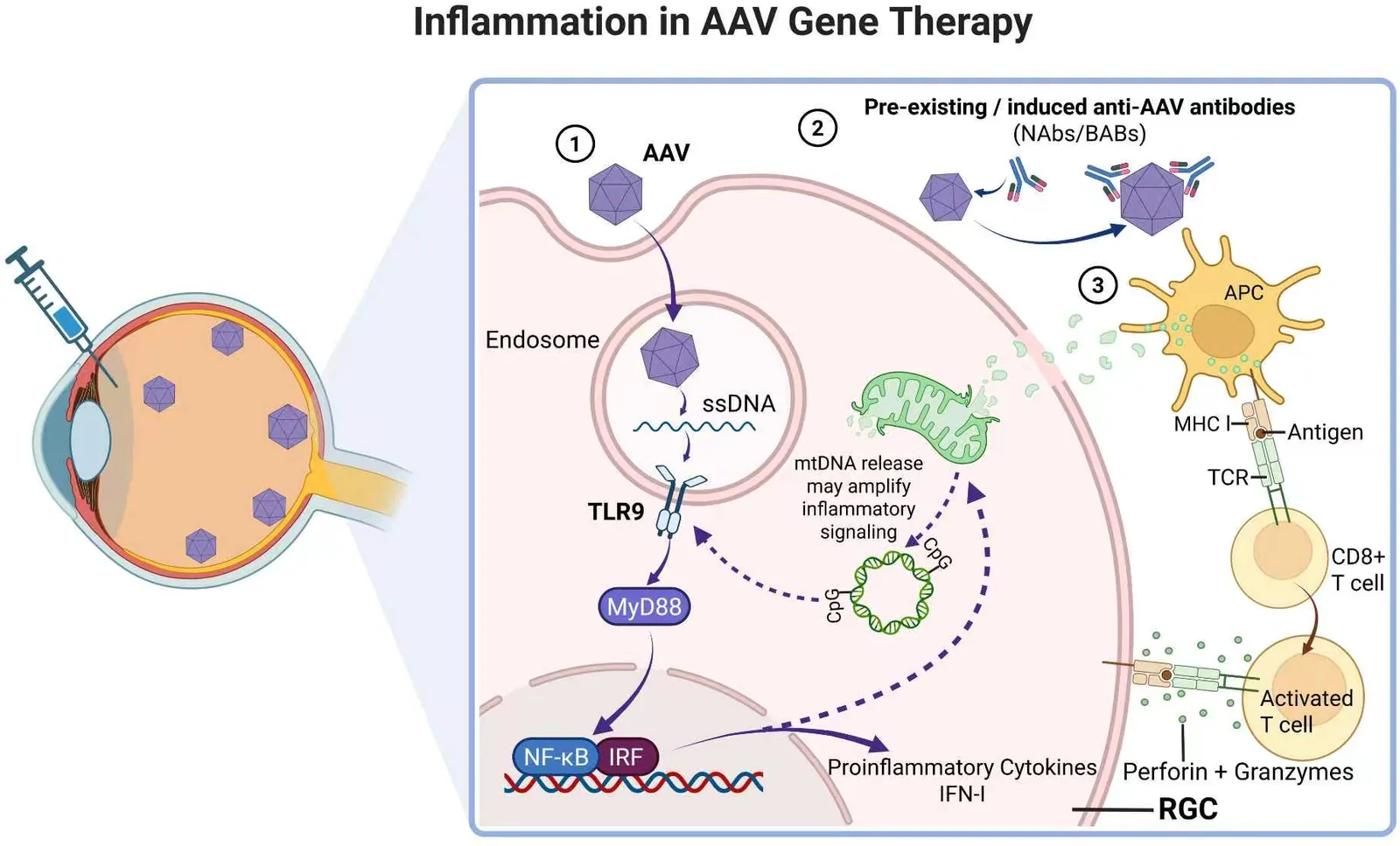
Proposed mechanisms of inflammation in AAV gene therapy for LHON. Following intravitreal delivery, AAV enters retinal cells through endocytic pathways, and CpG motifs within the vector genome may be sensed by TLR9 in endosomal compartments, leading to MyD88-dependent innate immune activation. In the setting of mitochondrial stress, endogenous mtDNA may further prime inflammatory signaling. In parallel, neutralizing antibodies may reduce transduction efficiency, and antigen presentation of capsid- or transgene-derived peptides may contribute to adaptive immune responses. Together, these mechanisms may contribute to intraocular inflammation and variable therapeutic efficacy. Source: Biorender.com.

### 4.4 Comparison with other ocular AAV programs

The inflammatory profile of lenadogene nolparvovec is most similar to that of other intravitreal AAV products. In the OPTIC study of intravitreal ixoberogene soroparvovec for neovascular age-related macular degeneration, anterior chamber and vitreous inflammation were dose dependent and responded to topical corticosteroids. Inflammation was more persistent at 6 × 10^11^ vg/eye than at 2 × 10^11^ vg/eye, although vision remained stable in both groups (85). This provides clinical evidence that reducing intravitreal vector dose can improve ocular tolerability without necessarily compromising efficacy.

Subretinal programs have shown a different pattern. In the phase 3 study of voretigene neparvovec for RPE65-associated retinal dystrophy, no deleterious immune response was identified, and most ocular adverse events were mild and related to vitrectomy or subretinal administration (86). Subretinal RGX-314 also produced no clinically recognized inflammation beyond that expected after vitrectomy, although pigmentary changes and vision loss occurred in one participant at the highest dose (87). A recent meta-analysis found higher pooled rates of anterior and intermediate or posterior uveitis after intravitreal than after subretinal AAV delivery (88). These comparisons should still be interpreted cautiously because route, capsid, dose, promoter, transgene, target cell, surgical procedure, and corticosteroid regimen differ across programs.

The available evidence supports three practical conclusions. First, the lowest dose that maintains adequate retinal ganglion cell transduction should be selected. Second, vector genome design and total capsid exposure should be considered together. Third, trials should use standardized inflammation grading and prespecified analyses linking inflammation to visual and structural outcomes. Corticosteroid prophylaxis remains reasonable, but current LHON data do not identify the regimen that provides the best balance between inflammation control and treatment burden. **Table 1** summarizes the strength and main limitations of the evidence for inflammatory mechanisms in LHON and AAV-related immune responses. This evidence provides the basis for the immunomodulatory strategies discussed in the next section.

**Table 1:** Qualitative evidence supporting inflammatory mechanisms in Leber hereditary optic neuropathy (LHON).

Evidence domain	Qualitative observation	Inflammatory interpretation	LHON specificity	Evidence strength	Key limitation	References
Clinical retinal imaging	Adaptive-optics studies have investigated cells with inflammatory-cell morphology in untreated LHON eyes.	Supports possible local retinal immune-cell recruitment or activation during LHON.	Direct human LHON evidence	Limited	Conference-level evidence; cell identity and causal relationship to retinal ganglion-cell loss remain unconfirmed.	(115)
Human pathology	Postmortem LHON optic nerve showed severe axonal depletion, fibrocytic scarring, degeneration debris, and minimal inflammation.	Provides direct histopathologic evidence that an inflammatory component can accompany active LHON axonal degeneration.	Direct human LHON evidence	Limited to moderate	Single postmortem case and late-stage tissue; temporal and causal relationships cannot be established.	(41)
LHON–multiple sclerosis overlap	LHON mitochondrial variants can coexist with inflammatory demyelinating disease, including white-matter lesions and cerebrospinal-fluid oligoclonal bands.	Indicates that mitochondrial vulnerability and adaptive neuroinflammation can interact in a clinically recognizable subgroup.	Direct but overlap-specific	Moderate	Rare phenotype; the association may largely reflect chance co-occurrence and should not be generalized to classical LHON.	(47, 116)
Complex I dysfunction and oxidative stress	LHON mutations impair complex I function and increase reactive oxygen and nitrogen species in cybrids and patient-derived cells.	Oxidative stress can activate redox-sensitive inflammatory signaling, including NF-κB programs.	Direct upstream LHON evidence; inflammatory step indirect	Moderate	Direct demonstration of downstream inflammatory signaling in human LHON retina or optic nerve remains limited.	(1, 117)
Mitophagy dysregulation	LHON cell and patient-derived models show disrupted mitochondrial quality control and abnormal clearance of dysfunctional mitochondria.	Persistence of damaged mitochondria may sustain reactive oxygen species and release mitochondrial damage-associated molecular patterns.	Direct LHON-model evidence; inflammatory consequence inferred	Moderate	Mitophagy phenotypes vary by model; inflammatory consequences have not been directly validated in human LHON tissue.	(7, 118)
Mitochondrial DNA sensing	Mitochondrial injury can release mitochondrial DNA into the cytosol or extracellular space.	Mitochondrial DNA can engage cGAS–STING and TLR9 pathways, inducing type I interferon and NF-κB programs.	Mechanistic extrapolation	Limited	No definitive demonstration of mitochondrial-DNA sensing in human LHON retina or optic nerve.	(12)
Inflammasome activation	Reactive oxygen species, oxidized mitochondrial DNA, and defective mitochondrial clearance are established triggers of NLRP3 activation in neurodegenerative settings.	NLRP3 activation could promote caspase-1 activity and maturation of IL-1β and IL-18, amplifying retinal ganglion-cell injury.	Mechanistic extrapolation	Limited	NLRP3 activation has not been directly validated in LHON retinal or optic-nerve tissue.	(119)
Microglial and astrocytic response to optic-nerve injury	Across optic-nerve injury models, activated microglia and astrocytes release cytokines, chemokines, complement components, and reactive oxygen species and participate in debris clearance.	Provides a biologically relevant mechanism for secondary inflammatory amplification after LHON retinal ganglion-cell axonal stress.	Indirect, disease-adjacent evidence	Limited to moderate	Responses are context- and time-dependent and may be neurotoxic, reparative, or both.	(96, 120)

Evidence grading: Strong, replicated direct evidence in human LHON tissue or patients; moderate, direct but limited human evidence or consistent LHON-model evidence with a plausible inflammatory link; limited, indirect evidence extrapolated from mitochondrial or optic-nerve injury models. These qualitative categories are intended for narrative synthesis and do not constitute a formal GRADE assessment.

## 5 Immunomodulatory strategies: future directions for combination therapy

Treatment for LHON has long focused on the bioenergetic defect caused by pathogenic mtDNA variants. This approach remains central, because complex I dysfunction is the primary driver of retinal ganglion cell vulnerability. Yet LHON progression is unlikely to be purely metabolic. Mitochondrial stress can promote innate immune activation, including cGAS–STING-related interferon signaling and NLRP3 inflammasome activation (10, 11). These pathways may amplify neuronal injury after the initial metabolic insult. This provides a rationale for combination therapy that supports mitochondrial function and, when appropriate, limits harmful inflammation.

Idebenone is the best-established metabolic treatment for LHON. Clinical evidence suggests that some patients benefit, especially when treatment is started within a suitable therapeutic window (89). Its main role is to improve electron transport and reduce oxidative stress. It is not designed to block downstream inflammatory signaling. AAV-mediated ND4 gene replacement offers a different strategy by addressing the genetic defect more directly. Clinical studies have shown encouraging visual outcomes, but intravitreal AAV exposure can trigger intraocular inflammation and immune responses (6, 88). For this reason, future treatment models should not view metabolic support, gene replacement, and immune control as separate strategies. They should be considered parts of one disease-stage and treatment-specific framework.

### 5.1 A Stage- and treatment-specific framework for immunomodulatory management in LHON

Immune management in LHON should be guided by disease stage and treatment context. In the acute or rapidly progressive phase, patients may show optic disc hyperemia and peripapillary retinal nerve fiber layer swelling. These findings can mimic optic neuritis and may lead to empirical corticosteroid treatment (3, 90). This is a common clinical problem, but LHON is not a typical demyelinating optic neuropathy. Corticosteroids should not delay mtDNA testing, genetic confirmation, or LHON-specific counseling. If corticosteroids are used, they should be limited to selected cases after careful exclusion of inflammatory, infectious, and demyelinating optic neuropathies (91–93). Their role in this setting is adjunctive and diagnostic-context dependent, not disease-specific.

In the chronic stage, the clinical picture is different. Acute swelling gives way to optic atrophy, and the inflammatory signal is likely to be lower grade. Experimental data support a role for retinal and optic nerve microglial activation in retinal ganglion cell injury (94, 95). This makes the microglia–inflammation axis a plausible target for neuroprotection (96). Still, this concept should be presented as a future direction rather than current standard care. Agents that suppress microglial activation or inflammatory amplification, such as minocycline, have shown protective effects in retinal or optic nerve injury models, but LHON-specific clinical evidence is still insufficient (97–100). Their possible value lies in reducing secondary injury, not replacing metabolic therapy.

More selective approaches may also be considered in future studies. The cGAS–STING and NLRP3 pathways are attractive targets because they link mitochondrial damage, cytosolic nucleic acid sensing, inflammasome activation, and cytokine release (10, 11, 101). Inhibition of these pathways could, in theory, reduce inflammatory amplification after mitochondrial stress. But several issues must be solved before clinical translation. These include the correct treatment window, the route of delivery, the dose needed for retinal and optic nerve exposure, and the risk of impairing host defense. Long-term suppression of innate immunity may increase infection risk or interfere with immune surveillance. For this reason, pathway-specific immunomodulation should first be tested in well-defined preclinical models and early-phase trials with structural, functional, and biomarker endpoints.

The periprocedural period of AAV gene therapy is the clearest current setting for immune management in LHON. In this context, the inflammatory trigger is defined: intraocular exposure to the viral vector, capsid proteins, vector genome, and transgene expression cassette. Published ND4-targeted trials show that ocular inflammation is common after intravitreal lenadogene nolparvovec, although most events are mild or moderate and respond to anti-inflammatory treatment (68, 70, 88). Immune management should therefore be built into AAV trial design. Local corticosteroids can be used as first-line treatment for anterior chamber or vitreous inflammation. Short systemic corticosteroid courses may be used when inflammation is more extensive or when a protocol requires prophylaxis. Pretreatment and tapering schedules may reduce early inflammatory risk, but the optimal regimen is still not defined (70, 102).

Future AAV protocols should also individualize immune management according to vector dose, delivery route, baseline ocular inflammation, prior AAV exposure, and systemic immune status (102–104). The goal is not to suppress immunity broadly. The goal is to control vector-related inflammation enough to preserve ocular safety and stable transgene expression. This distinction is important. Excessive immunosuppression may add risk without improving visual outcomes, while inadequate control may increase inflammation and reduce treatment tolerability.

Across the clinical course of LHON, immunomodulation should be used with a clear purpose. In the acute phase, the priority is correct diagnosis and careful avoidance of unnecessary steroid exposure. In the chronic phase, the main research goal is to test whether low-grade neuroinflammation can be safely reduced to limit secondary retinal ganglion cell injury. Around AAV gene therapy, immune control is already part of clinical protocol design and safety monitoring. Future trials should assess combination therapy with endpoints that include visual acuity, visual field, optical coherence tomography-based retinal nerve fiber and ganglion cell measures, and inflammatory fluid biomarkers. This approach would better capture the effects of metabolic support, gene replacement, and immune modulation than visual acuity alone (103, 105).

### 5.2 Lifestyle and environment: exogenous stressors and systemic metabolic homeostasis

Smoking is one of the clearest and most modifiable external risk factors in LHON. Epidemiologic studies support an association between tobacco exposure and visual conversion in mutation carriers; smoking cessation should therefore be routinely recommended (4). Tobacco smoke may increase oxidative stress and further compromise the limited mitochondrial reserve of the optic nerve (106). Heavy alcohol consumption and exposure to medications associated with mitochondrial toxicity or optic neuropathy, including ethambutol and linezolid, may likewise increase metabolic stress and should be addressed through clinical counseling and medication review (4, 107, 108). Long-term care in LHON should therefore include smoking cessation, alcohol restriction, and careful medication review and avoidance of potentially harmful agents whenever feasible.

As highly energy-dependent neurons, RGCs are particularly vulnerable to systemic metabolic stress (109). Severe fatigue, acute infection, or major psychological stress may increase ATP demand and, in the setting of mtDNA mutations, may further destabilize an already fragile energetic balance, thereby potentially contributing to visual deterioration (4, 110). Clinically relevant nutritional deficiencies should also be corrected when identified. By contrast, evidence supporting specific antioxidant dietary patterns or ketogenic interventions in LHON remains insufficient for clinical recommendations (111–113). These measures should be regarded as supportive risk-reduction strategies rather than established immunomodulatory treatments.

## 6 Conclusion

LHON should be viewed not only as a complex I-related bioenergetic disorder, but also as a condition in which mitochondrial stress and sterile immune signaling may together shape neuronal injury and treatment response. Pathogenic mtDNA variants disrupt oxidative phosphorylation and disturb mitochondrial homeostasis. In this setting, persistent mitochondrial stress may promote a low-grade pro-inflammatory state rather than acting only as a source of metabolic failure. Although direct *in vivo* evidence from LHON tissue remains limited, available studies suggest that mitochondrial dysfunction may increase the susceptibility of retinal cells to innate immune activation (3, 11, 114). This interaction may contribute to local inflammatory amplification in the retina and optic nerve and, in turn, to retinal ganglion cell degeneration.

Clinical observations also support a link between mitochondrial dysfunction and immune signaling in LHON. This has implications for both diagnosis and treatment. The LHON-MS overlap suggests that, in some patients, mitochondrial vulnerability may interact with susceptibility to neuroinflammation. In clinical practice, LHON should also be carefully distinguished from optic neuritis, as misclassification may lead to inappropriate management.

This issue has become more important with the development of intravitreal AAV-mediated ND4 gene therapy. Although the overall safety profile has been acceptable, intraocular inflammation remains common. This indicates that ocular immune privilege is relative and that immune management should be part of treatment planning. Experimental and clinical findings together suggest that pre-existing mitochondrial stress and vector-triggered immune responses may jointly increase inflammatory burden and influence treatment durability (72, 79). Future progress will likely require closer integration of strategies targeting both mitochondrial dysfunction and immune activation. These may include stage-specific immunomodulation, biomarker-guided monitoring, reduced vector immunogenicity, and better control of modifiable external stressors such as tobacco exposure and avoidable medications with mitochondrial toxicity.

Important gaps remain. Much of the current framework is still based on non-ocular systems or experimental models. These do not fully explain the selective vulnerability of retinal ganglion cells, the incomplete penetrance of LHON, or its clinical heterogeneity. Direct evidence from human tissue remains limited, and the timing of immune activation relative to mitochondrial injury is still unclear. Future work should focus on several priorities. More relevant translational models are needed to capture both mitochondrial dysfunction and ocular immune responses. Multicenter prospective cohorts with deep phenotyping are needed to better define disease heterogeneity. Structural, functional, and fluid biomarkers should also be integrated more systematically into interventional studies. Single-cell and spatial multi-omics may further improve disease stratification and help identify therapeutic windows. In parallel, biomarker-guided studies may improve patient selection and support efforts to reduce vector immunogenicity. Overall, progress in this field will likely require a broader framework that considers mitochondrial dysfunction together with innate immune and neuroinflammatory responses.

## Glossary

AAV: Adeno-associated virus
AI: Artificial intelligence
ATP: Adenosine triphosphate
ATF4: Activating transcription factor 4
BAK: BCL-2 antagonist/killer
BAX: BCL-2-associated X protein
BCVA: Best-corrected visual acuity
CD8: Cluster of differentiation 8
cGAMP: Cyclic guanosine monophosphate–adenosine monophosphate
cGAS: Cyclic GMP–AMP synthase
CLPP: Caseinolytic mitochondrial matrix peptidase proteolytic subunit
CNS: Central nervous system
CpG: Cytosine–phosphate–guanine
CRISPR: Clustered regularly interspaced short palindromic repeats
DAMPs: Damage-associated molecular patterns
DELE1: DAP3-binding cell death enhancer 1
eIF2α: Eukaryotic initiation factor 2 alpha
GRADE: Grading of Recommendations Assessment, Development and Evaluation
HLA: Human leukocyte antigen
HRI: Heme-regulated inhibitor
IFN-I: Type I interferon
IL-1β: Interleukin-1 beta
IL-18: Interleukin-18
iPSC: Induced pluripotent stem cell
IRF3: Interferon regulatory factor 3
LC3: Microtubule-associated protein 1 light chain 3
LC3B: Microtubule-associated protein 1 light chain 3B
LHON: Leber hereditary optic neuropathy
LHON–MS: Leber hereditary optic neuropathy–multiple sclerosis overlap
MERTK: MER proto-oncogene tyrosine kinase
MOMP: Mitochondrial outer membrane permeabilization
MRI: Magnetic resonance imaging
mPTP: Mitochondrial permeability transition pore
MS: Multiple sclerosis
mtDAMPs: Mitochondrial damage-associated molecular patterns
mtDNA: Mitochondrial DNA
MyD88: Myeloid differentiation primary response 88
NADH: Reduced nicotinamide adenine dinucleotide
ND4: NADH dehydrogenase subunit 4
NF-κB: Nuclear factor kappa B
NLRP3: NLR family pyrin domain-containing 3
OMA1: Overlapping activity with m-AAA protease 1
PINK1: PTEN-induced kinase 1
RGCs: Retinal ganglion cells
ROS: Reactive oxygen species
STING: Stimulator of interferon genes
TBK1: TANK-binding kinase 1
TLR9: Toll-like receptor 9
VDAC: Voltage-dependent anion channel
VDAC1: Voltage-dependent anion channel 1
vg: Vector genomes
WoSCC: Web of Science Core Collection
YME1L: YME1-like protein 1
